# Astaxanthin Promotes the Survival of Adipose-Derived Stem Cells by Alleviating Oxidative Stress via Activating the Nrf2 Signaling Pathway

**DOI:** 10.3390/ijms24043850

**Published:** 2023-02-14

**Authors:** Chang-Sheng Yang, Xiao-Shuang Guo, Ying-Ying Yue, Yu Wang, Xiao-Lei Jin

**Affiliations:** Maxillofacial Surgery Department 4 of Plastic Surgery Hospital, Chinese Academy of Medical Sciences & Peking Union Medical College, Beijing 100144, China

**Keywords:** fat grafting, mesenchymal stem cell, oxidative stress, astaxanthin, Nrf2 signaling pathway, apoptosis

## Abstract

The survival of free fat grafts is dependent primarily on adipose-derived stem cells (ADSCs); however, ADSCs are susceptible to oxidative stress in the recipient area. Astaxanthin (Axt) is a natural xanthophyll carotenoid with potent antioxidant properties and numerous clinical applications. To date, the therapeutic potential of Axt in fat grafting has not been explored. The purpose of this study is to investigate the effects of Axt on oxidatively stressed ADSCs. An oxidative model of ADSCs was developed to simulate the host’s microenvironment. Oxidative insult decreased the protein levels of Cyclin D1, type I collagen alpha 1 (COL1A1), and type II collagen alpha 1 (COL2A1), while increasing the expression of cleaved Caspase 3 and secretion of interleukin-6 (IL-6) and tumor necrosis factor-alpha (TNF-α) in ADSCs. Axt pre-treatment significantly reduced oxidative stress, increased the synthesis of an adipose extracellular matrix, alleviated inflammation, and restored the impaired adipogenic potential in the present model. Furthermore, Axt immensely activated the NF-E2-related factor 2 (Nrf2) pathway, and ML385, an inhibitor of Nrf2, could negate Axt’s protective effects. Additionally, Axt alleviated apoptosis by inhibiting bcl-2-associated X protein (BAX)/Caspase 3 signaling and improving the mitochondrial membrane potential (MMP), which could also be abolished by ML385. Our results suggest that Axt may exert its cytoprotective effect on ADSCs through the Nrf2 signaling pathway and could be therapeutic in fat grafting.

## 1. Introduction

Autologous fat grafting (AFG) involves harvesting fat tissue from a defined donor site and injecting it into the desired area to augment its appearance [1]. AFG has been greatly recognized and improved in plastic surgery due to its safety, easy availability, and versatility. The use of AFG is now commonplace in facial rejuvenation, breast surgery, and other aspects of aesthetic and reconstructive surgery. Moreover, fat grafting has shown its regenerative versatility and has been utilized to treat tough situations including chronic ulcers or scars, scleroderma, and Dupuytren contracture [2,3,4].

However, owing to hypoxia, oxidative stress, and mechanical injuries, the main concern in the application of AFG is its inconsistent and low graft take rate. Lv et al. demonstrated that the absorption rate after facial fat grafting varied from 17 to 74% and the pooled retention was 47% [5]. The low retention rate and the erratic nature of free fat grafts may largely be attributed to oxidative stress in the host microenvironment within 24 to 48 h after transplantation [6]. In a study conducted by Kerfant et al., the level of oxidative stress in adipose tissue was significantly higher one hour after fat collection as compared to the level immediately following liposuction [7]. Researchers also reported that oxidative insults decrease the proliferation, impair the differentiation competency, and exacerbate the apoptosis of ADSCs [8,9].

As a mediator of cellular response during an oxidative stress condition, the NF-E2-related factor 2 (Nrf2) signaling system plays an influential role in maintaining cellular homeostasis [10]. Under basal conditions, Nrf2 is sequestered by cytoplasmic Kelch-like ECH-associated protein 1 (Keap1) and remains inactive. However, in the case of oxidative stress, Nrf2 detaches from Keap1, translocates to the nucleus, and upregulates the expression of downstream proteins including heme oxygenase 1 (HO-1), glutathione peroxidase 4 (GPX4), and NADPH quinone oxidoreductase (NQO1).

Astaxanthin, a form of carotenoid referred to as xanthophyll, is a substance distributed widely in seafood such as salmon, crabs, or shrimp. Since being separated from lobsters in 1938 by Kuhn, R. et al., Axt has exhibited anti-oxidative, anti-inflammatory, anti-tumor, and cardioprotective properties [11]. Axt is a powerful scavenger of ROS and has superior anti-oxidative properties [12]. In addition, Axt has demonstrated the ability to protect cells against oxygen free radicals by inducing Nrf2-mediated antioxidant enzymes and this potent efficacy was observed at low Axt concentrations and with no toxicity observed [13,14].

To determine the efficacy of Axt in promoting ADSC survival in vitro, Axt was evaluated in a cellular oxidative model for its effect on adipogenic competency, extracellular matrix (ECM) synthesis, apoptosis, and adipogenic potential. Further investigation was conducted into the regulatory role of Nrf2 signaling in the cytoprotective effect of Axt.

## 2. Results

### 2.1. Axt Improved the Cell Viability and Proliferation of ADSCs

**Figure 1:**
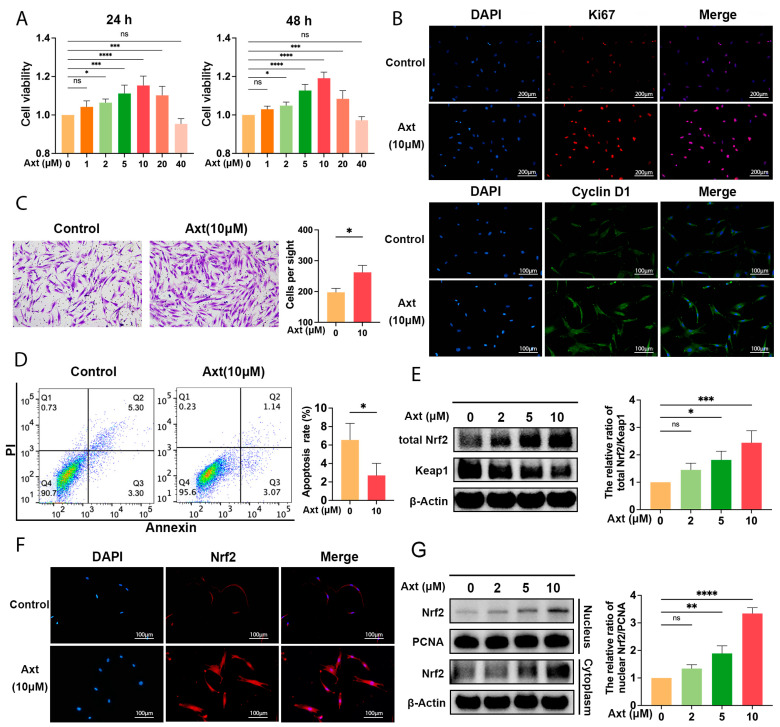
Axt improved cell activity and activated Nrf2 in human ADSCs. (**A**) The effect of Axt (1, 2, 5, 10, 20, and 40 μM) administration for 24 h and 48 h on ADSCs was evaluated by a CCK-8 Assay (*n* = 5); (**B**) Immunofluorescence staining of Ki67 (red, 100×, scale bar = 200 μm) and Cyclin D1 (green, 200×, scale bar = 100 μm) of ADSCs was performed to assess cell proliferation; (**C**) Transwell cell migration assay (100×). ADSCs were treated with vehicle (0 µM) or Axt (10 µM) for 36 h; (**D**) Apoptosis rates of ADSCs after 24 h Axt treatment were evaluated by Annexin V/PI staining through flow cytometry; (**E**) ADSCs were treated with Axt (2, 5, and 10 μM) for 24 h. The protein expression levels of Keap1 and Nrf2 were determined by western blot; (**F**) Immunofluorescence staining (red, 200×, scale bar = 100 μm) and western blot were used to demonstrate the translocation of Nrf2; (**G**) ADSCs were treated with Axt (2, 5, and 10 μM) for 24 h. The protein expression levels of nuclear and cytoplasmic Nrf2 were detected by western blot. Quantitative data were presented as the mean ± standard deviation (SD) (*n* = 3). * *p* < 0.05, ** *p* < 0.01, *** *p* < 0.001, **** *p* < 0.0001.

ADSCs were characterized by multilineage differentiation and a flow cytometry assay (Supplementary Materials Figure S1). Cell counting kit-8 (CCK-8) assays were conducted to evaluate the toxic effects of Axt at various concentrations (1, 2, 5, 10, 20, and 40 μM) on ADSCs (Figure 1A). For both the 24 h and 48 h Axt treatment, cell viability increased with the Axt concentration from 2 to 10 μM in a dose-dependent manner, while the cell viability of ADSCs treated with 20 and 40 μM Axt decreased with the increased concentration of Axt (24 h: *p* < 0.05 for 2 μM, *p* < 0.001 for 5 μM, *p* < 0.0001 for 10 μM, *p* < 0.001 for 20 μM, vs. the 0 μM; 48 h: *p* < 0.05 for 2 μM, *p* < 0.0001 for 5 μM, *p* < 0.0001 for 10 μM, *p* < 0.001 for 20 μM, vs. the 0 μM). Axt administration (10 μM) raised the expression of Ki67 and Cyclin D1 protein, implying that Axt could improve the proliferation of ADSCs (Figure 1B). Therefore, 10 μM was utilized as the optimal Axt concentration in the subsequent experiments.

### 2.2. Axt Promoted Migration and Did Not Induce Apoptosis

A Transwell cell migration assay was conducted to investigate the effect of Axt on migration. As illustrated in Figure 1C, under exposure to Axt (10 μM) for 36 h, the migration abilities of the cells were significantly enhanced compared with the control group (0 μM) (*p* < 0.05). The Annexin V/PI staining assay was performed to quantitatively analyze the apoptosis of ADSCs. As shown in Figure 1D, Axt treatment for 24 h did not exacerbate cell apoptosis. Although there was a reduction in the percentage of apoptotic cells (Q2 + Q3) in the 10 μM Axt treatment group compared to the 0 μM Axt group (*p* < 0.05, Figure 1D), the difference was not significant enough to illustrate that Axt reduced apoptosis.

### 2.3. Axt Induced the Activation of Nrf2 in ADSCs

Axt is a potent antioxidant and the transcription factor Nrf2 has been reported to affect the redox balance in ADSCs. The expression levels of Nrf2/Keap1 pathway proteins were determined in ADSCs. When Axt was administered at 2 μM, 5 μM, and 10 μM, there was a significant increase in total Nrf2/Keap 1 in a dose-dependent manner (*p* < 0.05 for 5 μM, *p* < 0.001 for 10 μM, vs. the 0 μM; Figure 1E). Furthermore, Axt exposure results in a remarkable nuclear translocation of Nrf2 (Figure 1F), and the ratio of nuclear Nrf2/proliferating cell nuclear antigen (PCNA) increased significantly with the increasing concentration of Axt (*p* < 0.01 for 5 μM, *p* < 0.0001 for 10 μM, vs. the 0 μM; Figure 1G). Based on the evidence, Axt is capable of elevating Nrf2 levels by inactivating its endogenous inhibitor, Keap1, and initiating the translocation of Nrf2 from the cytoplasm to the nucleus.

### 2.4. The Hydrogen Peroxide (H_2_O_2_)-Induced Oxidative Damage in ADSCs

**Figure 2:**
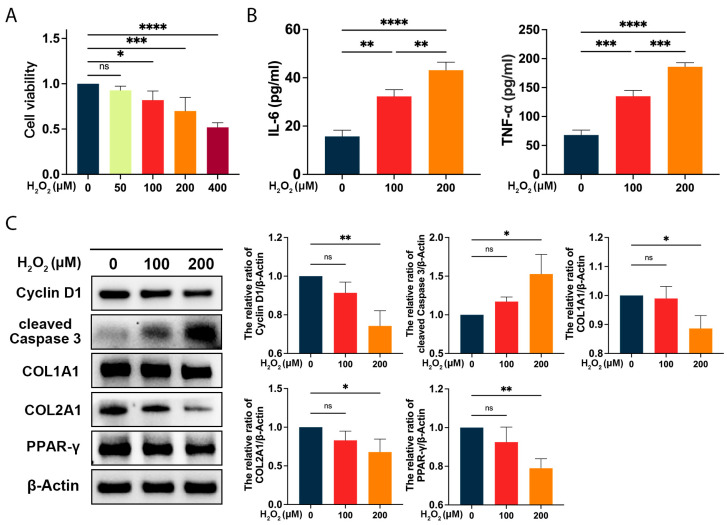
The hydrogen peroxide (H_2_O_2_)-induced oxidative stress compromised the cellular activities of ADSCs. (**A**) The toxic effect of H_2_O_2_ (50, 100, 200, and 400 μM) administration on ADSCs for 24 h was detected by CCK-8 assays; (**B**) The levels of IL-6 and TNF-α in the cell media of ADSCs after 24 h H_2_O_2_ treatment; (**C**) Western blot was used to demonstrate the protein expressions of Cyclin D1, cleaved Caspase 3, COL1A1, COL2A1, and PPAR-γ in ADSCs after 24 h 200 μM H_2_O_2_ treatment. Quantitative data were presented as the mean ± SD (*n* = 3). * *p* < 0.05, ** *p* < 0.01, *** *p* < 0.001, **** *p* < 0.0001.

H_2_O_2_ has been widely reported to exert oxidative insults in the literature [15]. To explore an optimal condition, we examined the impact of H_2_O_2_ on ADSCs at increasing concentrations. CCK-8 assays revealed that H_2_O_2_ concentration exceeding 100 μM compromised the cell viability (*p* < 0.05 for 100 μM, *p* < 0.001 for 200 μM, *p* < 0.0001 for 400 μM, vs. the 0 μM; Figure 2A). H_2_O_2_ significantly induced the secretion of IL-6 (*p* < 0.01 for 100 μM, vs. the 0 μM; *p* < 0.01 for 200 μM, vs. the 100 μM; Figure 2B) and TNF-α (*p* < 0.001 for 100 μM, vs. the 0 μM; *p* < 0.001 for 200 μM, vs. the 100 μM; Figure 2B). As demonstrated in Figure 2C, H_2_O_2_ downregulated the expression level of Cyclin D1 (*p* < 0.01 for 200 μM, vs. the 0 μM), COL1A1 (*p* < 0.05 for 200 μM, vs. the 0 μM), COL2A1 (*p* < 0.05 for 200 μM, vs. the 0 μM), and peroxisome proliferator-activated receptor-γ (PPAR-γ) (*p* < 0.01 for 200 μM, vs. the 0 μM), while increasing the protein level of cleaved Caspase 3 (*p* < 0.05 for 200 μM, vs. the 0 μM). In the present study, a 200 μM H_2_O_2_ treatment inhibited cell proliferation, decreased extracellular matrix secretion, induced apoptosis, and impaired adipogenic potential in ADSCs, and therefore this treatment was utilized in subsequent research.

### 2.5. Axt Protected ADSCs against Oxidative Insult In Vitro

**Figure 3:**
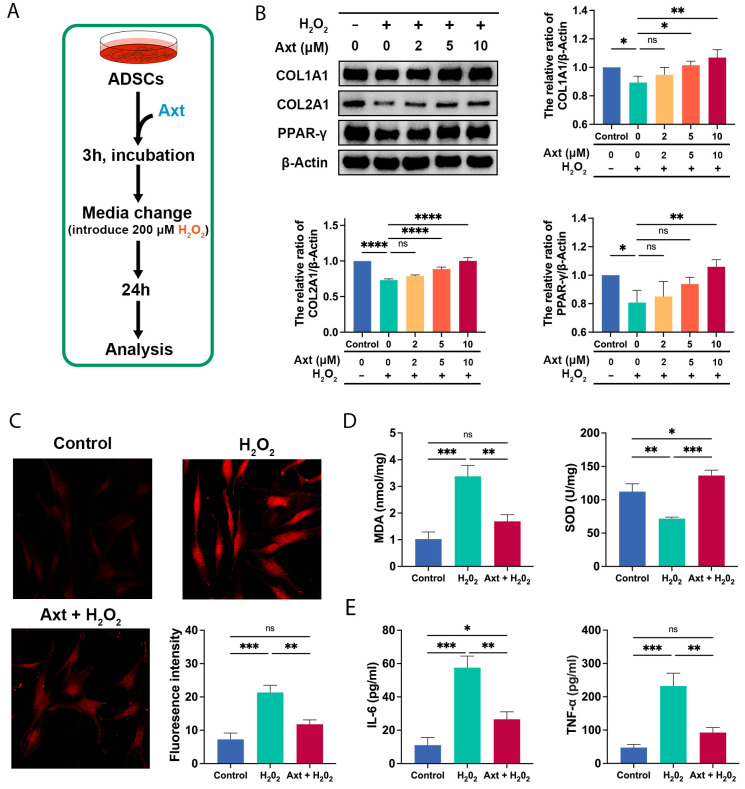
Cytoprotective effects of Axt on oxidatively stressed ADSCs in vitro. (**A**) A scheme for introducing Axt treatment and oxidative insults in ADSCs; (**B**) The expression levels of COL1A1, COL2A1, and PPAR-γ were evaluated by western blot; The concentration of Axt was 10 μM in the following experiments; (**C**) Intracellular ROS in ADSCs were assessed using dichloro-dihydro-fluorescein diacetate (DCFH-DA) staining followed by fluorescence microscopy. The magnification was 200×; (**D**) Measurements of MDA levels and SOD activities in ADSCs lysates; (**E**) The levels of IL-6 and TNF-α in the cell media of ADSCs. Quantitative data were presented as the mean ± SD (*n* = 3). * *p* < 0.05, ** *p* < 0.01, *** *p* < 0.001, **** *p* < 0.0001.

In the present model, ADSCs synthesized fewer COL1A1 and COL2A1, which are important components of the fat tissue extracellular matrix. We explored the potential protective effects of Axt, and as demonstrated in Figure 3B, Axt largely restored the decreased expression of COL1A1 (*p* < 0.05 for 5 μM, *p* < 0.01 for 10 μM, vs. the 0 μM) and COL2A1 (*p* < 0.0001 for 5 μM, *p* < 0.0001 for 10 μM, vs. the 0 μM) in a dose-dependent manner. Our results indicated that Axt could reverse the adipogenic potential loss of ADSCs by upregulating PPAR-γ (*p* < 0.01 for 10 μM, vs. the 0 μM, Figure 3B). Moreover, Axt relieved the oxidative stress by eliminating reactive oxygen species (ROS) content (*p* < 0.01 for Axt + H_2_O_2_, vs. the H_2_O_2_, Figure 3C), reducing malondialdehyde (MDA) levels (*p* < 0.01 for Axt + H_2_O_2_, vs. the H_2_O_2_, Figure 3D), and increasing reactive oxygen species (SOD) activity (*p* < 0.001 for Axt + H_2_O_2_, vs. the H_2_O_2_, Figure 3D). The ELISA assay demonstrated that Axt pre-treatment significantly reduced the secretion of IL-6 (*p* < 0.01 for Axt + H_2_O_2_, vs. the H_2_O_2_, Figure 3E) and TNF-α (*p* < 0.01 for Axt + H_2_O_2_, vs. the H_2_O_2_, Figure 3E). The results illustrate the protective effects of Axt on oxidatively stressed ADSCs.

### 2.6. Axt Safeguarded ADSCs against Oxidative Stress Via Nrf2 Signaling

**Figure 4:**
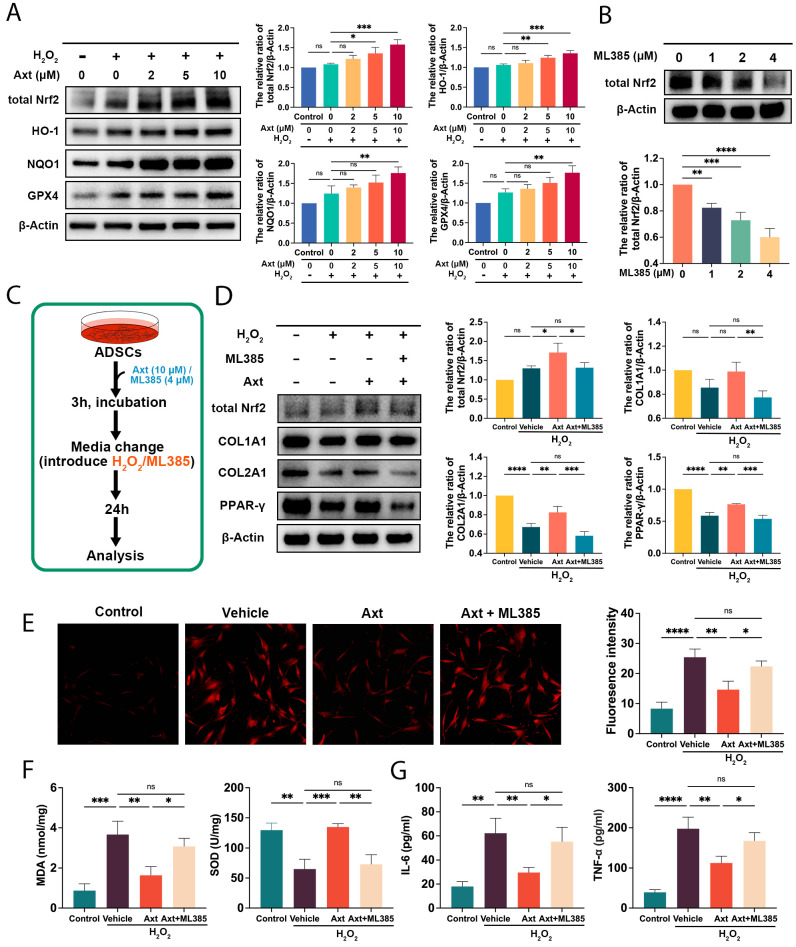
Nrf2 pathway-mediated protective effects of Axt on oxidative-stressed ADSCs. (**A**) Western blot was used to detect the expression levels of total Nrf2, HO-1, NQO1, and GPX4 in ADSCs; (**B**) After 3 h treatment of ML385 at different concentrations, the protein expression of total Nrf2 was detected by western blot to demonstrate the optimal concentration; (**C**) A scheme for introducing Axt, ML385, and oxidative insults to ADSCs; 10 μM Axt and 4 μM ML385 were utilized in the following experiments; (**D**) The protein expression of total Nrf2, COL1A1, COL2A1, and PPAR-γ was measured via western blot to verify the role of the Nrf2 signaling pathway in Axt-induced cytoprotective effects; (**E**) Intracellular ROS in ADSCs were determined using DCFH-DA staining followed by fluorescence microscopy. The magnification was 100×; (**F**) MDA and SOD levels were measured in ADSCs lysates; (**G**) IL-6 and TNF-α in culture media were quantified by ELISA. Quantitative data were presented as the mean ± SD (*n* = 3). * *p* < 0.05, ** *p* < 0.01, *** *p* < 0.001, **** *p* < 0.0001.

Oxidative stress is detrimental to ADSCs’ survival and differentiation. The cytoprotective effect of Axt is achieved through the enhancement of Nrf2 signaling. This study examines the role of Nrf2 signaling in oxidatively stressed ADSCs. As displayed in Figure 4A, the administration of Axt resulted in dramatically upregulated levels of Nrf2 (*p* < 0.05 for 5 μM, *p* < 0.001 for 10 μM, vs. the 0 μM) and subsequent downstream genes including HO-1 (*p* < 0.01 for 5 μM, *p* < 0.001 for 10 μM, vs. the 0 μM), NQO1 (*p* < 0.01 for 10 μM, vs. the 0 μM), and GPX4 (*p* < 0.01 for 10 μM, vs. the 0 μM). To determine the protective effects of Nrf2, 4 μM ML385 was used to obviate the function of Nrf2 (Figure 4B). The results in Figure 4D illustrate that the inhibition of Nrf2 eliminated the Axt-induced restoration of COL1A1 (*p* < 0.01 for Axt + ML385, vs. the Axt), COL2A1 (*p* < 0.001 for Axt + ML385, vs. the Axt), and PPAR-γ (*p* < 0.001 for Axt + ML385, vs. the Axt). Moreover, the data in Figure 4E–G demonstrate that ML385 abolished the safeguard effects on ADSCs in the ROS content (*p* < 0.05 for Axt + ML385, vs. the Axt), MDA level (*p* < 0.05 for Axt + ML385, vs. the Axt), SOD activity (*p* < 0.01 for Axt + ML385, vs. the Axt), IL-6 level (*p* < 0.05 for Axt + ML385, vs. the Axt), and TNF-α secretion (*p* < 0.05 for Axt + ML385, vs. the Axt). These findings support the hypothesis that Nrf2 protects ADSCs against the imbalance of cellular redox.

### 2.7. Axt Alleviated the H_2_O_2_-Induced ADSC Apoptosis and Mitochondrial Injury

**Figure 5:**
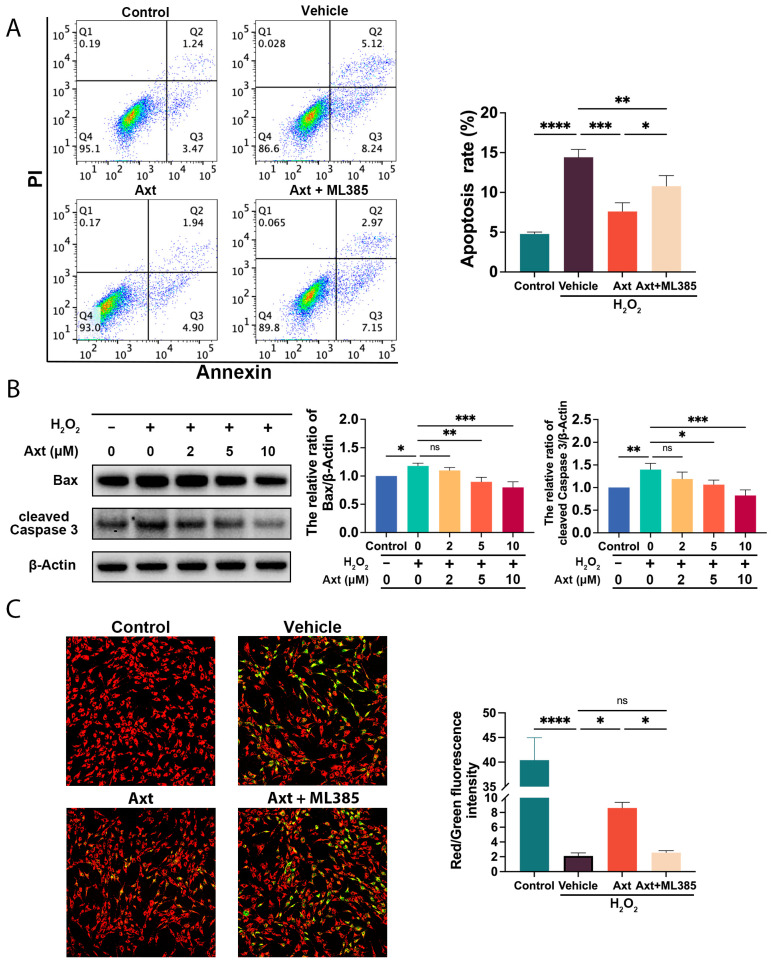
Effects of Axt on H_2_O_2_-induced apoptosis and mitochondria injury. (**A**) ADSCs were stained with Annexin V/PI to demonstrate apoptosis via flow cytometry; 10 μM Axt and 4 μM ML385 were utilized; (**B**) The expression levels of BAX and cleaved Caspase 3 were determined by Western blot; (**C**) The mitochondrial membrane potential of ADSCs was detected by JC-1 staining and quantified; 10 μM Axt and 4 μM ML385 were used. Quantitative data were presented as the mean ± SD (*n* = 3). * *p* < 0.05, ** *p* < 0.01, *** *p* < 0.001, **** *p* < 0.0001.

As illustrated in Figure 5A, the qualitative measurement of ADSC apoptosis by Annexin V-FITC/PI staining showed that a 10 μM Axt treatment significantly reduced apoptosis (*p* < 0.001 for Axt, vs. the vehicle), and this effect could be reversed by 4 μM ML385 (*p* < 0.05 for Axt + ML385, vs. the Axt). Moreover, we also detected the classic BAX/Caspase 3 signaling and demonstrated that Axt inhibited the expression of BAX (*p* < 0.01 for 5 μM, *p* < 0.001 for 10 μM, vs. the 0 μM) and cleaved Caspase 3 (*p* < 0.05 for 5 μM, *p* < 0.001 for 10 μM, vs. the 0 μM) in a dose-dependent manner (Figure 5B). Since mitochondria are one of the most important targets of BAX [16], we investigated the mitochondrial membrane potential (MMP) with JC-1 staining (Figure 5C). Axt (10 μM) restored the MMP in ADSCs that had been exposed to oxidative stress (*p* < 0.05 for Axt, vs. the vehicle), and ML385 (4 μM) could cancel this effect (*p* < 0.05 for Axt + ML385, vs. the Axt). These results indicate that Axt could downregulate BAX/Caspase 3 signaling to resist mitochondrial injuries via Nrf2 signaling.

## 3. Discussion

The fat grafting procedure has been introduced for over a century and has remained a weighty component of the plastic surgeon’s arsenal. However, surgeons continue to question its efficacy due to the high absorption rate [17,18]. After transplantation, non-vascularized adipose tissue grafts are susceptible to oxidative and ischemic stress, resulting in the inconsistent survival of stromal cells within 24 h [19,20]. Without achieving optimal fat retention, AFT cannot be considered a superior technique.

Free fat graft transplantation relies heavily on ADSCs [21]. Based on Eto et al.’s graft replacement theory, almost all adipocytes, except those located within 300 μm of the periphery, undergo necrosis within the first day [6]. Activated ADSCs promote angiogenesis, while dead adipocytes form lipid droplets that are phagocytosed by macrophages. New adipocytes are generated from progenitor cells in the regenerating zone to replace dead adipocytes. Fat survival volume depends on the number of adipocytes that are successfully replaced by ADSCs. In addition, several studies have reported that stem cells are more susceptible to free radical damage than other cells [22]. Furthermore, oxidative stress results in cellular senescence and death, and this effect cannot be reversed by eliminating the oxidant [9]. The effects of oxidative stress on ADSCs should therefore be considered in the application of AFT.

Axt is a powerful natural antioxidant that is known to exert multiple functions, including anti-inflammation, anti-oxidation, and anti-apoptosis ones [23]. Axt has therefore been used in a variety of clinical scenarios, including skin rejuvenation, neurodegeneration, and cancer [24,25,26]. We examined the safety of Axt under physiological conditions and found that cell viability was significantly enhanced. The increased fluorescence intensity of Ki67 and Cyclin D1 compared with the control group suggests that Axt promotes ADSCs proliferation. Additionally, Axt promotes cell migration and did not induce apoptosis in ADSCs. As a result of these findings, we can conclude that Axt concentrations under 20 μM are safe and promote the cell activities of ADSCs.

In the present study, we developed an oxidative stress model induced by hydrogen peroxide to mimic the host oxidative stress microenvironment after fat grafting. H_2_O_2_ has been widely used to induce cellular redox imbalance in epidermal cells, retinal ganglion cells, and bone-marrow-derived stem cells [27,28,29]. For ADSCs, a 200 μM H_2_O_2_ treatment for 24 h is sufficient to affect their functions and activities but is not lethal. Therefore, 200 μM H_2_O_2_ was utilized in the following research.

We evaluated the cell status of ADSCs by measuring the expression levels of Cyclin D1, cleaved Caspase 3, COL1A1, COL2A1, and PPAR-γ. The downregulation of Cyclin D1 and elevation of cleaved Caspase 3 indicate inhibited proliferation and stimulated apoptosis in the present model. Type I and Type II collagen are two important proteins in adipose ECM, providing the complex scaffold to sustain the function and structure of stromal cells [30,31]. A decrease in the synthesis of COL1A1 and COL2A1 in ADSCs suggests that oxidative stress affects adipose ECM remodeling [32]. PPAR-γ is concerned with the differentiation and maintenance of mature adipocytes and is therefore critical in the formation of adipocytes [33,34]. In our model, the protein expression of PPAR-γ was significantly reduced, which indicated the loss of adipogenic competency in ADSCs. Moreover, increasing concentrations of IL-6 and TNF-α in the culture media depict an inflammatory microenvironment [35].

Further investigation was conducted to determine whether Axt had cytoprotective effects in the present model. In response to Axt treatment, the cell activities of ADSCs were preserved in a dose-dependent manner under oxidative stress. Axt stimulated the synthesis of COL1A1 and COL2A1 and upregulated the expression of PPAR-γ which indicates that Axt could improve ECM deposition and restore impaired adipogenic potential. Previous researchers have reported the ability of Axt to inhibit lipogenesis in 3T3-L1 cells [36,37], including suppressing lipid accumulation and downregulating lipogenesis-related genes including PPAR-γ. The discrepancy among our studies may mainly be attributed to different cell species, cellular models, and Axt treatments. Biological markers of oxidative damage include ROS, MDA, and SOD, reflecting an antioxidative capability [38,39,40]. By eliminating ROS, reducing MDA activity, and increasing SOD levels, Axt alleviated oxidative stress. In addition, Axt mitigated inflammation by suppressing the secretion of IL-6 and TNF-α.

The Nrf2 signaling pathway plays a pivotal role in modulating cellular redox status [41]. We demonstrated that Axt triggered the activation of Nrf2 under physiological conditions. In the present model, Axt secured Nrf2 from the sequestration of Keap1, induced the translocation of Nrf2 from the cytoplasm to the nucleus, and elevated the expression of downstream antioxidant proteins, including HO-1, NQO1, and GPX4. Subsequently, to confirm that the cytoprotective effects of Axt were mediated by Nrf2, we inhibited the expression and function of Nrf2 through ML385, a specific Nrf2 inhibitor [42]. The ML385 treatment led to a significant reduction in Nrf2 expression and abolished the protective effects of Axt. According to our findings, Axt decreases oxidative stress, promotes the synthesis of ECM, and mitigates inflammation by activating Nrf2 signaling.

Axt also exerted an anti-apoptosis feature via Nrf2 signaling in our study. We observed that Axt remarkably inhibited the expression level of BAX and cleaved Caspase 3. BAX/Caspase 3 is a classic signaling pathway that regulates apoptosis, in which BAX is a pro-apoptotic signaling molecule, whereas Caspase-3 is an effector in the final stages of apoptosis [43,44]. The activation and translocation of BAX to mitochondria result in a rapid loss of MMP, which triggers the activation of the pro-apoptotic caspase cascade [45,46]. JC-1 staining demonstrated that Axt could restore MMP via Nrf2 signaling. In conclusion, Axt is capable of downregulating BAX/Caspase 3 signaling via the Nrf2 pathway to prevent mitochondrial injury.

As far as we are aware, this is the first time that Axt has been employed to improve ADSCs’ survival under oxidative stress. Axt improved the viability, proliferation, and immigration of ADSCs. Accordingly, in the ADSC oxidative model, pretreatment with Axt preserved the ECM deposition and adipogenic potential, reduced the production of pro-inflammatory cytokines, and inhibited apoptosis via Nrf2 signaling. We should note, however, that the clinical samples in this study were obtained from Asian middle-aged females (45.4 ± 5.413 years old). Senescence inhibits cell proliferation, decreases the differentiation potential, raises oxidative stress, and reduces the paracrine activity of ADSCs [47,48,49]. There is a possibility that senescence may be an additional factor that exacerbates the impact of oxidative stress on ADSCs. It would be beneficial to examine the protective role of Axt in younger samples. Moreover, although ML385 is an efficacious inhibitor, there was still an expression of Nrf2; the silence of the NRF2 gene may make our results more persuasive. Additionally, we were unable to explain how Axt activates Nrf2 signaling and how the Nrf2 pathway modulates ADSC inflammation. Lastly, we still need to examine the effectiveness of Axt in an animal model.

## 4. Materials and Methods

### 4.1. Reagents

Astaxanthin and ML385 were obtained from MCE (Princeton, NJ, USA) and were dissolved in 0.1% dimethyl sulfoxide (DMSO, Sigma–Aldrich, Darmstadt, Germany). Hydrogen peroxide was supplied by Aladdin (Shanghai, China). Phosphate-buffered saline (PBS), fetal bovine serum (FBS), bovine serum albumin (BSA), Penicillin-Streptomycin-Neomycin (PSN) Antibiotic Mixture, and Minimum Essential Medium-Alpha (α-MEM) were obtained from Gibco (Waltham, MA, USA).

### 4.2. Human Adipose-Derived Stem Cell Isolation, Cultivation, and Characterization

Patients (5 female donors, 45.4 ± 5.413 years old) from Plastic Surgery Hospital, Chinese Academy of Medical Sciences, and Peking Union Medical College (CAMS & PUMC) were recruited to provide healthy adipose tissues following selective abdominal liposuction. All donors signed informed consent forms. After being washed twice with PBS, fat tissues were digested in a shaker at 37 °C for 45 min with the same volume of 0.2% (*w*/*v*) type I collagenase (Sigma-Aldrich, Darmstadt, Germany). Upon centrifugation at 400× *g* for 5 min, the supernatant was discarded, and the precipitation was resuspended in α-MEM. The cell suspension was filtered through a 70 μm nylon filter and then cultured in complete α-MEM (containing 10% FBS and 1% PSN) on a 10 cm culture plate. ADSCs were cultured at 37 °C in a humidified atmosphere of 5% CO_2_, and the media were changed every three days. At 80% confluence, the cells were defined as passage 1 and re-plated. Passages 2–3 were used for the following experiments. ADSCs were characterized by multilineage differentiation (Cyagen Bioscience, Guangzhou, China) and analysis of the expression of CD73, CD90, CD105, CD34, CD11b, CD19, and CD45 (BD Biosciences, Franklin Lakes, NJ, USA) using a BD Biosciences flow cytometer and Flow Jo software (version 10.8.1, BD Biosciences, Franklin Lakes, NJ, USA).

### 4.3. Cell Counting Kit-8 (CCK-8) Assay

The viability of ADSCs was tested using a cell counting kit-8 purchased from Dojindo laboratories (Kumamoto-ken, Japan) in accordance with the manufacturer’s protocol. In brief, cells were seeded on a 96-well plate at a concentration of 5000 per well, and then 10 μL CCK-8 solution was added to each well. After 1.5 h of incubation at 37 °C without exposure to light, the absorbance was measured using a microplate reader (PerkinElmer, Waltham, MA, USA) at 450 nm. The experiment was repeated five times.

### 4.4. Immunofluorescence Microscopy

ADSCs were seeded in a glass bottom dish. Following treatment, the cells were washed twice with cold PBS before being fixed in 4% paraformaldehyde for 20 min. The dishes were incubated with PBS solution containing 0.1% triton-X and 5% BSA for 60 min at room temperature to permeabilize and block nonspecific binding sites. After discarding the solution, cells were incubated with primary antibodies overnight at 4 °C. Primary antibodies against Ki67 (Abcam, Cambridge, UK, 1:300), Cyclin D1 (Proteintech, Rosemont, IL, USA, 1:250), and Nrf2 (Abcam, Cambridge, UK, 1:150) were utilized. After being washed with PBS 3 times, the dishes were incubated with fluorescent conjugate secondary antibodies for 1 h at room temperature without light. Anti-Mouse secondary conjugated with Alexa Fluor 488 (Abcam, Cambridge, UK) and Anti-Rabbit secondary conjugated with Alexa Fluor 647 (Abcam, Cambridge, UK) were used. Cell nuclei were stained with DAPI (Beyotime, Shanghai, China) for 10 min, and images were obtained using a BX53 upright fluorescence microscope (Olympus, Tokyo, Japan).

### 4.5. Cell Migration Assay

A Transwell assay (Corning, Corning, NY, USA) was performed to assess the migration capacity of ADSCs. Passage 2–3 cells were collected and resuspended at a density of 6 × 10^4^ cells in 200 µL of the serum-free α-MEM and seeded in the upper compartment of the chamber. Then, 600 µL of complete α-MEM containing vehicle or AXT was added to the lower chamber. After incubation for another 36 h, the chambers were removed, and cotton swabs were used to remove the cells on the upper surface of the membrane. ADSCs that had migrated into the microporous membrane were washed twice with PBS, fixed with methanol solution for 30 min, and stained with 0.1% crystal violet (Beyotime, Shanghai, China) for 20 min. Finally, a microscope (Olympus, Tokyo, Japan) was used to observe the cells.

### 4.6. Western Blot

After washing twice with cold PBS, the cells were lysed in RIPA lysis buffer (Applygen, Beijing, China) containing Protease Phosphatase Inhibitor Cocktail (Beyotime, Shanghai, China). Afterward, the cells were scraped and centrifuged at 12,000× *g* for 10 min at 4 °C. The supernatants were collected after centrifugation. Nuclear and cytoplasmic proteins were extracted using a Nuclear and Cytoplasmic Protein Extraction Kit (Beyotime, Shanghai, China). Protein concentrations were assessed using a BCA assay kit (Beyotime, Shanghai, China). A sample of 20 μg protein was separated by SDS-PAGE (4–20%), and the bands were transferred to a PVDF membrane (Sigma-Aldrich, Darmstadt, Germany). After blocking with 5% skim milk for 2 h at room temperature, membranes were incubated with primary antibodies overnight at 4 °C. Primary antibodies against Nrf2 (Abcam, Cambridge, UK, 1:1000), Keap 1 (Proteintech, Rosemont, IL, USA, 1:1000), PCNA (Proteintech, Rosemont, IL, USA, 1:1500), β-Actin (Abcam, Cambridge, UK, 1:5000), Cyclin D1 (Proteintech, Rosemont, IL, USA, 1:5000), Caspase 3 (Abcam, Cambridge, UK, 1:1000), COL1A1 (Abcam, Cambridge, UK, 1:4000), COL2A1 (Proteintech, Rosemont, IL, USA, 1:2000), PPAR-γ (Abcam, Cambridge, UK, 1:1000), HO-1 (Proteintech, Rosemont, IL, USA, 1:1000), NQO1 (Abcam, Cambridge, UK, 1:1000), GPX4 (Proteintech, Rosemont, IL, USA, 1:2000), and BAX (Proteintech, Rosemont, IL, USA, 1:3000) were employed in the experiments. The membrane was then washed 3 times and incubated with the appropriate secondary antibodies for 1 h at room temperature. The secondary antibodies used in the present study are as follows: HRP-conjugated Affinipure Goat Anti-Mouse IgG (H + L) (Proteintech, Rosemont, IL, USA, 1:4000) and HRP-conjugated Affinipure Goat Anti-Rabbit IgG (H + L) (Abcam, Cambridge, UK, 1:4000). The protein bands were visualized using BeyoECL Plus detection reagent (Beyotime, Shanghai, China), and band images were obtained via the Chemi-Doc Imaging System (Bio-Rad, Hercules, CA, USA). Semi-quantification of the bands was performed with ImageJ software (version 1.8.0, National Institutes of Health, Bethesda, MD, USA).

### 4.7. Cell Apoptosis Detection Assay

Cell apoptosis was analyzed by the FITC Annexin V Apoptosis Detection Kit purchased from BD Biosciences (Franklin Lakes, NJ, USA), and the procedures followed the manufacturer’s protocol. Cells were analyzed via a BD Biosciences flow cytometer (Franklin Lakes, NJ, USA), and the results were analyzed using FlowJo software (Version 10.8.1, Franklin Lakes, NJ, USA).

### 4.8. Enzyme-Linked Immunosorbent Assay (ELISA)

The secretion of IL-6 and TNF-α in the cell media was assessed by ELISA. ADSCs were seeded at the concentration of 2 × 10^4^ cells/cm^2^ on 6-well plates and incubated with 2 mL complete α-MEM per well. According to the manufacturer’s instructions, cell media were harvested, centrifuged, and analyzed for pro-inflammatory cytokines using the IL-6 (Abcam, Cambridge, UK) and TNF-ELISA kits (Abcam, Cambridge, UK). The concentrations of cytokines were determined by measuring absorbance at 450 nm using a multimode plate reader (PerkinElmer, Waltham, MA, USA), and each group was prepared in triplicate.

### 4.9. Reactive Oxygen Species (ROS) Detection

Intracellular ROS detection was performed using a Dichloro-dihydro-fluorescein diacetate (DCFH-DA) assay kit (Beyotime, Shanghai, China). Assays were conducted according to the manufacturer’s instructions and the cells were observed under a fluorescence microscope and analyzed using Image J software (version 1.8.0, National Institutes of Health, Bethesda, MD, USA).

### 4.10. Detection of Malonaldehyde (MDA) and Superoxide Dismutase (SOD)

The supernatant of the cell lysate was tested for the presence of MDA and the activity of SOD using commercial kits purchased from Beyotime (Shanghai, China). After lysing ADSCs with RIPA lysis buffer, the cells were scraped and centrifuged at 12,000× *g* for 10 min at 4 °C. The supernatants were collected for detection after centrifugation. Assays were performed according to the manufacturer’s protocols. Total protein concentration was determined using the BCA method.

### 4.11. Mitochondrial Membrane Potential (MMP) Detection

The mitochondrial membrane potential was evaluated using a JC-1 detection kit (Solarbio, Beijing, China). JC-1 staining was performed on cultured cell monolayers following the manufacturer’s protocols. The results were observed under a fluorescence microscope and analyzed using Image J software (version 1.8.0, National Institutes of Health, Bethesda, MD, USA).

### 4.12. Statistical Analysis

In the present study, all experiments were performed in triplicate except for the CCK-8 assay, which was performed five times. The cell viability and protein expression levels of the control group were regarded as standards, and the results of other groups were calculated as the percentages of the control group. The data were analyzed using GraphPad Prism 9 (Version 9.4.0, GraphPad Software, San Diego, CA, USA). For quantitative data, the Student’s *t*-test was utilized for the comparison of differences between two groups, and the one-way ANOVA followed by Bonferroni’s multiple comparison test was used for the comparison of more than two groups. The data were displayed as mean ± standard deviation (SD). *p* values < 0.05 were defined as statistical significance.

## 5. Conclusions

According to our results, Axt pretreatment could promote ECM synthesis, restore adipogenic potential, inhibit inflammation, and regulate the apoptosis of ADSCs under oxidative stress via the Nrf2 signal pathway. Axt may be an effective treatment for improving the survival rate of autologous fat grafts.

## Data Availability

Not applicable.

## Supplementary Materials

The following supporting information can be downloaded at: https://www.mdpi.com/article/10.3390/ijms24043850/s1.

## Abbreviations

ADSCs	adipose-derived stem cells
Axt	Astaxanthin
Nrf2	NF-E2-related factor 2
COL1A1	type I collagen alpha 1
COL2A1	type II collagen alpha 1
IL-6	interleukin-6
TNF-α	tumor necrosis factor-alpha
AFT	autologous fat grafting
Keap1	Kelch-like ECH-associated protein 1
BAX	bcl-2-associated X protein
HO-1	heme oxygenase 1
GPX4	glutathione peroxidase 4
NQO1	NADPH quinone oxidoreductase
CCK-8	cell counting kit-8
PPAR-γ	peroxisome proliferator-activated receptor-γ
MDA	malondialdehyde
SOD	reactive oxygen species
SOD	superoxide dismutase
ECM	extracellular matrix
DCFH-DA	dichloro-dihydro-fluorescein diacetate
