# Supplementary material for: Astaxanthin Promotes the Survival of Adipose-Derived Stem Cells by Alleviating Oxidative Stress via Activating the Nrf2 Signaling Pathway
Source: Int J Mol Sci. 2023 Feb 14;24(4):3850. doi: 10.3390/ijms24043850 (PMC9959672; doi:10.3390/ijms24043850)
Supplement: Supplementary file 1 [file ijms-24-03850-s001.zip › ijms-2176789-supplementary.pdf]

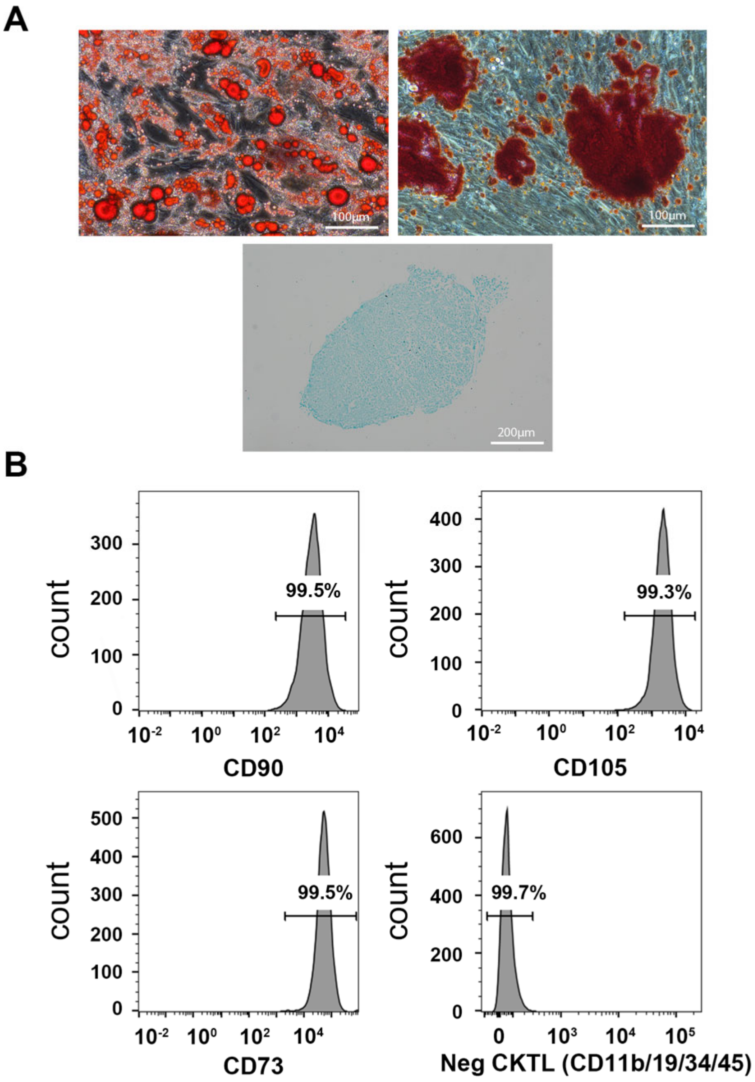

Figure S1. Characterization of ADSCs differentiation and surface markers. ADSCs were induced into adipogenic, osteogenic, and chondrogenic lineages. (A) Adipogenic differentiation was stained with Oil Red O, and the magnification was 200X. (scale bar = 100 μm); Osteogenic differentiation was stained with Alizarin Red S, and the magnification was 200X. (scale bar = 100 μm); Chondrogenic differentiation was stained with Alcian Blue, and the magnification was 100X. (scale bar = 200 μm); (B) Flow cytometry analysis of ADSCs specific surface markers expression. Cells were positive for CD90(99.5%), CD105(99.3%), CD73(99.5%) and negative (0.3%) for CD11b, CD19, CD34 and CD45.
